# Security Analysis and Improvements to the PsychoPass Method

**DOI:** 10.2196/jmir.2366

**Published:** 2013-08-13

**Authors:** Bostjan Brumen, Marjan Heričko, Ivan Rozman, Marko Hölbl

**Affiliations:** ^1^Institute of InformaticsFaculty of Electrical Engineering and Computer ScienceUniversity of MariborMariborSlovenia

**Keywords:** security, passwords, cryptanalysis

## Abstract

**Background:**

In a recent paper, Pietro Cipresso et al proposed the PsychoPass method, a simple way to create strong passwords that are easy to remember. However, the method has some security issues that need to be addressed.

**Objective:**

To perform a security analysis on the PsychoPass method and outline the limitations of and possible improvements to the method.

**Methods:**

We used the brute force analysis and dictionary attack analysis of the PsychoPass method to outline its weaknesses.

**Results:**

The first issue with the Psychopass method is that it requires the password reproduction on the same keyboard layout as was used to generate the password. The second issue is a security weakness: although the produced password is 24 characters long, the password is still weak. We elaborate on the weakness and propose a solution that produces strong passwords. The proposed version first requires the use of the SHIFT and ALT-GR keys in combination with other keys, and second, the keys need to be 1-2 distances apart.

**Conclusions:**

The proposed improved PsychoPass method yields passwords that can be broken only in hundreds of years based on current computing powers. The proposed PsychoPass method requires 10 keys, as opposed to 20 keys in the original method, for comparable password strength.

## Introduction

In a recent paper, El Emam, Moreau, and Jonker highlighted the importance of using strong passwords to protect personal health information in clinical trials stored in files [1]. In their settings, we have a typical offline password guessing scheme.

Pietro Cipresso et al have commented on the paper by elaborating on the potential problem people may have creating passwords that are complex but at the same time easy to remember, and they propose a solution called the PsychoPass method [2]. The method is discussed in the context of user-protected files; however, it can also be used in other settings requiring a password, including administrator account passwords.

The proposed solution by Cipresso et al has some limitations to be considered. But before we describe the limitations, we present a short discussion on strong passwords, as both papers have omitted an explanation of why the passwords can be weak and how serious the weaknesses can be.

First, the general rule is that the strength of passwords is proportional to their length and to the type of symbols being used, provided that the password’s symbols are drawn randomly from a pool of possible symbols—the key concept here is randomness [3]. The formula expressing the number of possible combinations is *s*=*b* ^*le*^, where *s* is the total number of combinations, *b* is the total number of symbols in a domain and *le* is the length of the password in number of characters. Typically, the domain where the password symbols are drawn from consists of lowercase (a…z) and uppercase letters (A…Z), numbers (0…9) and special symbols (eg, “#$%&/). In English, the number of letters is 52 (26 lower case + 26 upper case), in addition to 10 numbers (0…9) and some symbols (eg, 13), totaling 75. If, for example, up to 7 characters are used for a password, there is a total of *s*=75^1^+75^2^+75^3^ +…+75^7^=75^8^-1 = 1,001,129,150,390,625 combinations. However, an adversary may try out all the combinations in a so-called brute force attack. Today, the reported speeds (*v*) are in the range of *v*=10^9^ combinations/second [4-7], up to 10^12^ combinations/second [3], for restoring a plaintext password from a given hash value, as was the scenario in the paper by El Emam et al [1]. The same scenario—an attack using precalculated hash values for cracking passwords generated by the PsychoPass method—can be used.

To find out how much time it takes to check all the combinations (in a worst-case scenario where the sought-after password is the very last one to try), we use the well-known equation from high-school physics, *t=s/v*, where time (the object has traveled) is the distance divided by velocity, which is in our case the number of combinations divided by the speed of how many combinations could be tested in 1 second. The calculation is based upon an assumption that the hash values of all possible combinations of letters are precomputed: *t*=*s*/*v*=1,001,129,150,390,625 comb. / 1,000,000,000 comb. / second)=1,001,129.15 seconds=16,685.49 minutes=278.09 hours=11.59 days. That is, it will take at most 11.59 days (11 days, 14 hours, 5 minutes, 29 seconds) to find the password. On average however, the time is halved (5 days, 19 hours, 2 minutes, 45 seconds).

Due to Moore’s Law [8] still in effect, the computing power doubles roughly every 18 months. In 10 years, we can expect the speeds to be 100 times faster than today’s speed. One should note, however, that the estimate of the increase of speeds based on Moore’s law is rather conservative. The techniques and algorithms advance faster than brute computing power (eg, using GPUs and server farms for hire [9]). Also, quantum computing will have an enormous impact on security [10].

Tables 1 and 2 list the number of all possible combinations for passwords of length up to 7 and 9, respectively, for different sizes of character pools (25=lower case letters only, 50=lower and upper case letters, 60=letters and numbers, 75=letters, numbers, and special symbols), the time required to check *all* possible combinations at today’s speeds, and the time it will take in 10 years from now due to speed improvements.

The tables describe a well-known phenomenon in the information security field: what is safe today will most probably not be safe tomorrow [3]. Consider the setting described in the original paper by El Emam et al [1], where personal health information in clinical trials is stored in files. These same files will be around for years and although an attacker might not have enough computing power today, he or she will have it a few years from now. If the files contain interesting medical data about a celebrity or a public person, for example, the disclosure will be as damaging in the future as it might be today.

Second, the general rule—the more symbols in a password the better—has an important exception: if the password is otherwise long, but is a word, it is considered to be a weak password (the characters are no longer drawn randomly, but from a specific distribution). Such a password is susceptible to a dictionary attack [3]. Suppose an adversary composes a dictionary of all words of all languages and calculates the corresponding hash value. While it is hard to tell how many languages there are in the world—the estimates vary from 5000 to 10,000 languages—there are 6909 living human languages catalogued [11]. Let us suppose that each language contains 1 million words—a recent study [12] estimated the number of words in the English lexicon was 1,022,000 in 2000. Based on these numbers, we estimate that the total number of human words would not exceed 7,060,998,000 words; the actual number is much lower due to overlapping of words between languages. It would take very little time for an adversary to try all the words as passwords: *t*=*s*/*v*=7,060,998,000 words / 1,000,000,000 words /s=7.06 seconds.

Today, dictionaries containing words and passwords have several billion entries [13,14]. When choosing the correct length of a password, it is essential to observe one of the basic principles of security [3]: (1) a password scheme is said to be computationally secure if the cost of breaking the cipher exceeds the value of the protected information, or (2) the time required to break the password exceeds the useful lifetime of the information. Today’s costs for building a password-cracking machine are negligible [4,6,7], so the only principle to rely on is the time required for breaking the password. For the purpose of this paper, let us assume that the useful lifetime of the (medical) information is 60 years. Under this assumption, a safe password would be made of at least 9 characters from upper and lowercase letters, numbers, and symbols.

The aim of this paper is to examine the strength of the PsychoPass method in light of these assumptions.

**Table 1:** Number of combinations of passwords of length up to 7 and maximum time to crack them now and in 10 years.

Size	No. of combinations	Time to crack now	Time to crack in 10 years from now
25	1,52588E+11	2 minutes, 33 seconds	0 seconds
50	3,90625E+13	10 hours, 51 minutes, 2 seconds	39 seconds
60	1,67962E+14	1 day, 22 hours, 39 minutes, 22 seconds	2 minutes, 48 seconds
75	1,00113E+15	11 days, 14 hours, 5 minutes, 29 seconds	16 minutes, 41 seconds

**Table 2:** Number of combinations of passwords of length up to 9 and maximum time to crack them now and in 10 years.

Size	No. of combinations	Time to crack now	Time to crack in 10 years from now
25	9,53674E+13	1 day, 2 hours, 29 minutes, 27 seconds	1 minutes, 35 seconds
50	9,76563E+16	3 years, 35 days, 6 hours, 44 minutes, 10 seconds	1 day, 3 hours, 7 minutes, 36 seconds
60	6,04662E+17	19 years, 63 days, 9 hours, 36 minutes	6 days, 23 hours, 57 minutes, 42 seconds
75	5,63135E+18	178 years 207 days, 16 hours, 17 minutes, 51 seconds	65 days, 4 hours, 15 minutes, 51 seconds

## Security Issues of the PsychoPass Method

**Figure 1:**
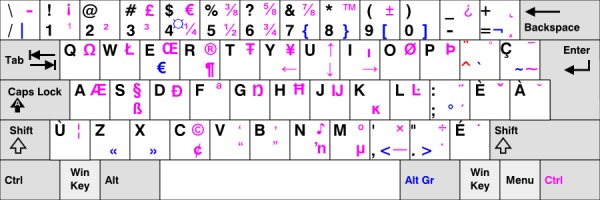
Canadian multilingual standard keyboard layout.

**Figure 2:**
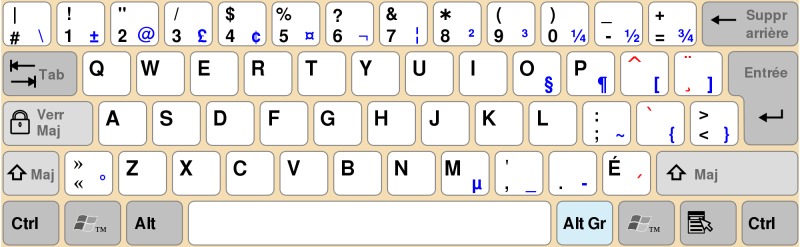
Canadian French keyboard layout.

We have identified two issues with the PsychoPass method proposed by Cipresso et al. First, their method works only on keyboards with the same layout. In many countries (eg, in Canada), there are several different keyboard layouts, rendering their method practically useless. For example, a sequence on a Canadian Multilingual Standard keyboard (see Figure 1) starting with key “w”, followed by combination “SHIFT” + key “3” produces password “w#”, while the same sequence repeated on a Canadian French keyboard (Figure 2) produces password “w/”.

**Figure 3:**
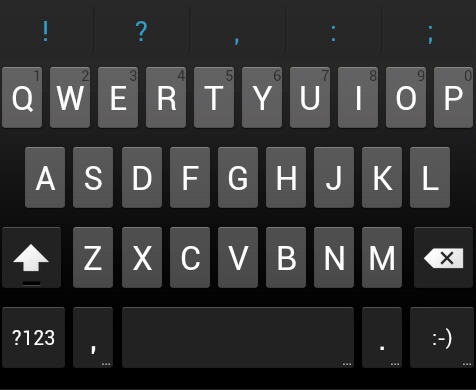
Android ICS keyboard layout.

The situation gets worse when the keyboards are of different types (QWERTZ vs QWERTY vs AWERTY) or when the method is used across various platforms, eg, from desktop PCs or desktops to mobile devices, such as Android-powered tablets (see layout in Figure 3 vs Figures 1 and 2).

The idea of PsychoPass is that a password can be created, memorized, and recalled by just thinking of an action sequence instead of a word or string of characters [2]. When the keyboard layout is different, the user cannot reproduce the very same password as she or he only knows the sequence of the keys, but not the key values themselves.

The above-mentioned problem is merely technical and requires the user to use the same type of keyboard. Additionally, with some basic training or professional help, a user can change the keyboard layout without physically replacing the keyboard. With a different system layout, a user would again reproduce the correct password even on the physically different keyboard. The interoperability between traditional and mobile devices remains a minor challenge.

Second, and more importantly, the method proposed by Cipresso et al has a security design issue because it produces predictable passwords, being prone to brute force attack. The PsychoPass method, when implemented as demonstrated by the authors in their video (and as can be seen from the figures and described in the paper) [2], produces a password such that it starts at a certain key and then proceeds only to the first neighbor of that key, and then again only to the first neighbor of that key, until the sequence length is reached; the password sequence is then repeated. Characters produced by such a procedure are not drawn randomly but are drawn according to some function (in this case, by the proximity function). As can be seen from the Cipresso et al’s Figure 1, they have produced a sequence “f-t-6-t-y-g-r-5”, where each key in the sequence is a neighbor to the previous one.

Proposing the use of adjacent keys on a keyboard produces combinations that are not only nonrandom, but these combinations themselves form a dictionary of finite combinations. As the PsychoPass method is publicly disclosed, constructing an algorithm for a dictionary-based attack script for the PsychoPass method is exceedingly easy.

The total number of different combinations (*s*) using the demonstrated PsychoPass method is *s=n*_*k*_ *•* ^*ble*^ *•n*_*s*_ *,* where *n*_*k*_ is the number of different characters on the keyboard from where the sequence can start, *b* is the number of possible next keys, *le* is the length of the produced sequence, and *n*_*s*_ is the number of repeated sequences.

At the beginning, we have some 45 key combinations (*n*_*k*_=45) for selecting the key as the starting point (the authors chose key “f”). From there on, each keyboard key has (at most) 8 neighbors (plus the key itself), so in each step only 1 out of 9 combinations (*b*=9) can be used. The authors have created a 24-key password by repeating the same sequence of length 8 (*le*=8) three times (*n*_*s*_=3) and have claimed that the password is a strong one. However, their claim is optimistic. The total number of different password combinations that can be produced by their method is *s*=*n*_*k*_ *•* ^*ble*^ *•n*_*s*_=45*•*9^8^•3=5,811,307,355. All such passwords can be checked in less than 6 seconds. Table 3 lists the amount of time required to test all passwords created by the PsychoPass method for a different number of sequence repetitions and a different number of keystrokes in a sequence.

It can be observed that the number of repetitions of sequences (*n*_*s*_) does not contribute significantly to the overall strength of the password. The only contributing factor is the number of letters in the password. For the proposed PsychoPass method to be considered safe and to produce strong password for today’s use, a user would have to remember a 17-key sequence. But when considering Moore’s law, a 20-key nonrepeating sequence should be used.

**Table 3:** Strength of original PsychoPass method for different parameter settings.

n_s_	le=8	le=17	le=20
1	2 seconds	23 years, 291 days, 46 minutes, 16 seconds	173 years, 176 days, 4 hours, 10 minutes, 57 seconds
2	4 seconds	47 years, 217 days, 1 hour, 32 minutes, 33 seconds	346 years, 352 days, 8 hours, 21 minutes, 53 seconds
3	6 seconds	71 years, 143 days, 2 hours, 18 minutes, 49 seconds	520 years, 163 days, 12 hours, 32 minutes, 50 seconds
4	8 seconds	95 years, 69 days, 3 hours, 5 minutes, 6 seconds	693 years, 339 days, 16 hours, 43 minutes, 46 seconds
5	10 seconds	118 years, 360 days, 3 hours, 51 minutes, 22 seconds	867 years, 150 days, 20 hours, 54 minutes, 43 seconds
6	12 seconds	142 years, 286 days, 4 hours, 37 minutes, 39 seconds	1040 years, 327 days, 1 hours, 5 minutes, 39 seconds
7	14 seconds	166 years, 212 days, 5 hours, 23 minutes, 55 seconds	1214 years, 138 days, 5 hours, 16 minutes, 36 seconds
8	15 seconds	190 years, 138 days, 6 hours, 10 minutes, 12 seconds	1387 years, 314 days, 9 hours, 27 minutes, 33 seconds

## Proposed Improvements to the PsychoPass Method

The PsychoPass method for generating passwords is based on a very interesting concept. The original version, which has a security issue, can be improved as follows. First, one should use the SHIFT key and ALT-GR key in combination with other keys. This way, the initial number of combinations (*n*_*k*_) increases from 45 to some 110 (=45 characters without shift + 45 characters with shift + some 20 characters with ALT-GR).

Second, a user should remember the next key that is not only 1 distance away, but 1 or 2 (or more). For example, if “Q” is initially selected, then “E” (or “e”), should be taken into the account as well. Here, “E” has a distance of 2 units from “Q”. This way the number of possible next keys increases from 9 to approximately 18 (approximately because it depends on the location of the key, eg, “B” has less two-unit distance neighbors than “E” since “B” is next to the space bar).

Now, combining the use of SHIFT and ALT-GR keys and the use of a larger distance between the keys increases the base (*b)* from 9 to approximately 54 (ie, 9 neighbors, each in combination with the plain key, SHIFT + key or ALT-GR + key). Combined, the total number of different passwords that can be produced by the improved method with 3 repetitions and sequence length of 9 characters is *s=n*_*k*_ *•* ^*ble*^ *•n*_*s*_ *=*110*•*54^9^•3=1,288,420,951,063,403,520.

All these passwords can be checked in 40 years, 312 days, 6 hours, 42 minutes, and 31 seconds. This is a considerable improvement over the 6-second attack on the original method.

**Table 4:** Strength of improved PsychoPass method for different parameter settings (truncated to years).

n_s_	le=8	le=9	le=10
1	0	13	735
2	0	27	1470
3	0	40	2206

Table 4 lists the time required (in years) to check all the password combinations under different parameter settings. It can be observed (again) that the sequence length is the key contributing factor to the overall security of the password. For the proposed improvement to the PsychoPass method to be considered safe and to produce strong password for today’s use, a user would have to remember a 10-key sequence, repeated only once.

## Conclusion

The PsychoPass method, as proposed by Pietro Cipresso et al in [2], has two issues. The first issue is merely a technical one: the passwords can be produced and reproduced only on keyboards with the same keyboard layout. The second issue is a security weakness: although the produced password is 24 characters long, the password is still weak. The weakness comes from the fact that the characters are not being drawn randomly but are based on proximity of keys on a keyboard. The passwords are not resilient to brute force attack, unless the repetition of the sequences is omitted and the length of the nonrepeating sequence is at least 20 characters. Such a requirement in the length raises a question of whether the purpose of the method—a sequence that is easy to remember—is still met.

We proposed an improvement to the PsychoPass method. First, the user needs to consider the use of the SHIFT and ALT-GR keys in combination with other keys. Second, the keys used need to be 1 or 2 distances apart (not only 1), and third, the number of keys in the sequence shall be at least 9, preferably 10. With the sequence length of 10 characters, there is no need to repeat the sequence as the repetition does not significantly improve the security of the total password. The improved PsychoPass method yields passwords that can only be broken in hundreds of years, considering the current computing powers. The proposed version requires 10 keys, as opposed to 20 keys in the original method, for comparable password strength.

## Abbreviations

AZERTY: specific keyboard layout for the characters of the Latin alphabet that takes its name from the first 6 letters to appear on the first row of alphabetical keys. It is mostly used in France and Belgium.
GPU: graphics processing unit
ICS: Ice Cream Sandwich (Android Version 4.0)
QWERTY: keyboard layout that is mostly used in United Kingdom, United States of America, Canada (and other countries) and is named after the first 6 letters in the first letter row on some keyboards.
QWERTZ: widely used keyboard layout in Central Europe. The name comes from the first 6 letters at the top left of the keyboard.
